# Tick HRF-dependent ferroptosis pathway to promote tick acquisition of *Babesia microti*

**DOI:** 10.3389/fcimb.2025.1560152

**Published:** 2025-03-12

**Authors:** Songqin Chen, Shanming Hu, Yongzhi Zhou, Jie Cao, Houshuang Zhang, Yanan Wang, Jinlin Zhou

**Affiliations:** Shanghai Veterinary Research Institute, Chinese Academy of Agricultural Sciences, Shanghai, China

**Keywords:** *Babesia microti*, ferroptosis, ticks, HRF, infection

## Abstract

*B. microti* is a tick-transmitted zoonotic erythrocytic intracellular parasite. Ferroptosis is an iron-dependent form of programmed cell death that affects pathogen replication in the host. Currently, there is limited research concerning the effect of tick ferroptosis on *Babesia* infection and the underlying mechanism of action. The present study used a *B. microti* -mouse- *Haemaphysalis longicornis* infection model in which nymphs fed on the blood of *B. microti*-infected mice. The midgut divalent iron (*p<0.01*) and reactive oxygen species (ROS) (*p<0.05*) levels were significantly elevated in infected ticks, and transmission electron microscopy (TEM) showed that mitochondrial ridges were absent or decreased in size. Downregulation of ferritin 1 and glutathione peroxidase 4 (GPX4) in ticks infected with *B. microti* suggests that these changes promote ferroptosis. *In vivo* studies demonstrated that the ferroptosis promoter Erastin increased *B. microti* load (*p<0.05*), while the inhibitor Ferrostatin-1 effectively decreased load (*p<0.01*). Tick histamine-releasing factor (HRF), a protein related to the antioxidant system, was downregulated in infected nymphs compared with uninfected nymphs (*p<0.05*), and interference with HRF promoted tick acquisition of *B. microti* (*p<0.001*). Transcriptomic analyses showed that HRF interference promotes tick ferroptosis by downregulating ferritin 1 and GPX4. Meanwhile, interference with tick HRF molecules showed increased divalent iron and ROS and decreased mitochondrial ridges compared with controls. These findings highlight the critical role of tick HRF molecules in regulating ferroptosis and acquisition of *B. microti*, thereby providing important insights for a deeper understanding of the tick-*Babesia* interaction.

## Introduction

Ticks, as blood-feeding arthropods, are vectors for a wide range of pathogens that pose significant threats to human health, including *Borrelia burgdorferi*, *Rickettsia rickettsii*, tick-borne encephalitis virus, and *Babesia* (McCoy et al., 2013; Brown Marusiak et al., 2022; Köhler et al., 2023). They can survive extreme environmental conditions by entering a state of metabolic dormancy (Parola and Raoult, 2001). Throughout their life cycle, ticks undergo four developmental stages and three feeding cycles, with a particular dependence on blood during the larval and nymphal stages, during which they can increase in size by nearly 100-fold (Perner et al., 2016). *Haemaphysalis longicornis*, also known as the Asian long-horned tick, is widely distributed in East Asia, Australia, New Zealand, and several Pacific islands, where it uses mammals as hosts and is capable of carrying at least 30 human pathogens, including *Babesia* (Tsuji et al., 2008; Breuner et al., 2020; Zhao et al., 2021). The interaction between *Babesia* and ticks involves intricate molecular mechanisms, wherein *Babesia* modulates the physiological state of ticks to facilitate its reproduction, while ticks utilize their innate immune system to counteract and suppress *Babesia* infection (Antunes et al., 2017; Bajer and Dwużnik-Szarek, 2021). However, the detailed molecular interactions involved in this relationship remain to be thoroughly investigated.

The tick histamine-releasing factor (HRF), also known as translationally controlled tumor protein, is a highly conserved protein present in all eukaryotes. It is involved in many biological processes, including apoptosis, autophagy, and protecting cells from oxidative stress damage (Lucibello et al., 2011; Bommer, 2017; Bommer and Telerman, 2020). A reduction in HRF molecules significantly affects tick blood feeding and decreases the transmission of *Borrelia burgdorferi* from ticks to their hosts (Dai et al., 2010). Our laboratory previously identified that tick HRF is involved in expressing ferritin 1, reducing reactive oxygen species (ROS) production, and lowering malondialdehyde levels by sequestering excess divalent iron cations (Sun et al., 2024). High levels of divalent iron enhance intracellular lipid peroxidation, leading to the accumulation of lipid peroxides, which in turn causes membrane disruption, cellular dysfunction, and ferroptosis (Song et al., 2021; Tian et al., 2023). Ferroptosis, a novel form of programmed cell death closely related, is intricately linked to iron metabolism and oxidative stress, influencing organismal growth, development, and pathogenic infections (Tang et al., 2021; Hu et al., 2023; Kim et al., 2023). Key anti-ferroptosis molecules include ferritin 1 and glutathione peroxidase 4 (GPX4), with GPX4 functioning as an antioxidant enzyme that reduces lipid peroxides, thereby protecting cell membranes from damage (Hou et al., 2016; Fuhrmann et al., 2020; Liu et al., 2023). Research on the interactions between ferroptosis and pathogens has shown that reduced ferritin 1 levels increase ROS production in mosquitoes, which impedes dengue virus infection (Zhu et al., 2019). Furthermore, inducing ferroptosis in the liver has been found to effectively reduce Plasmodium infection (Kain et al., 2020). These findings suggest that modulating the ferroptosis pathway may have a potential inhibitory effect on pathogen infection. However, there is a lack of studies examining the relationship between HRF and ferroptosis in ticks and their consequent influence on pathogen infection.

In the present study, we demonstrate that *B. microti* induces tick ferroptosis by downregulating the expression of ferritin 1 and GPX4 through the inhibition of HRF in ticks, which results in increased concentrations of divalent iron and elevated levels of reactive oxygen species (ROS). This process was further investigated *in vivo* using the ferroptosis promoter Erastin and the inhibitor Ferrostatin-1, revealing that ferroptosis in ticks promotes the acquisition of *B. microti*. Taken together, our results confirm the critical role of ferroptosis in *B. microti* infection and underline the importance of HRF in regulating tick ferroptosis.

## Materials and methods

### Ethics statement

All procedures for animal care and handling required for this experiment were approved by the Institutional Animal Care and Use Committee of the Shanghai Veterinary Research Institute (IACUC Approval No. SHVRI-SV-20230616-03, and SHVRI-20230602-01), and animal experiments were conducted following the Guidelines on the Humane Treatment of Laboratory Animals after approval.

### *Babesia*, ticks, and animals

The *B. microti* strain (ATCC PRA-99TM; Manassas, VA, USA) was kept in our laboratory and was obtained and maintained by serial passaging in BALB/c mice. Female BALB/c mice aged 5–6 weeks (18–20 g/mouse) were purchased from Suzhou Sibifu Biotechnology Co., Ltd (Suzhou, China) for *B. microti* passaging and tick infection experiments. The rabbits were provided by the Shanghai Laboratory Animal Center (Shanghai Institutes for Biological Sciences, Chinese Academy of Sciences) and were used to generate *H. longicornis* in the laboratory. The ticks were placed in the dark at 25°C and a relative humidity of 60%.

### Tick manipulation and infection studies

Tick infection with *B. microti* followed a previous laboratory study (Wu et al., 2017). In brief, when *B. microti* infection in mice reached 10–15%, nymphs that had molted for 3–6 weeks were allowed to feed on the mice (n = 60) to ensure that the tick’s rapid blood-feeding phase coincided with the peak of *B. microti* infection. Engorged ticks infected with *B. microti* were collected to characterize the presence of the pathogen in ticks, with 10 mice used in both the experimental and control groups. Giemsa staining was utilized to detect *Babesia microti* infection in mouse blood samples. Erythrocytes from a *B. microti*-infected mouse (50% infection rate) were lysed according to the manufacturer’s instructions (Tiangen, China) and incubated on ice for 5 min. After filtering through a 5 μm membrane, the *B. microti* precipitate was collected by centrifugation at 12,000 rpm for 10 min and resuspended in 500 μL sterile PBS. A 10 μL aliquot was mixed with Trypan Blue for counting and viability assessment using a BIO-RAD TC20 cell counter (4–6 μm range).

### Nucleic acid isolation and PCR

RNA was extracted using the TRIZOL method according to the manufacturer’s instructions (Invitrogen, USA) and converted to first-strand cDNA using a HiScript III RT SuperMix for qPCR (+gDNA wiper) kit (Vazyme Biotech, China). The cDNA was used to analyze the relative quantitative changes in gene expression (**Supplementary Table S1**). Samples were subjected to qRT-PCR using ChamQ Universal SYBR qPCR Master Mix (Q711, Vazyme) in a QuantStudio™5 Real-Time PCR System (Applied Biosystems™, New York, USA), and all samples were analyzed with three replicates. Elongation factor-1 (ELF1A, GenBank registry number AB836665) (Nijhof et al., 2009) was selected as an internal control for relative gene expression (following the 2^−ΔΔCt^ method).

### RNAi

Ticks were gene-RNA interference (RNAi) according to previously published methods using the primers listed in **Supplementary Table S2**. Interference-specific primers were designed based on laboratory transcriptome gene sequences of *HRF*, *GPX4*, and *Fe1*, while *Luciferase* was used as a control, and T7 RNA polymerase promoter sequences were added to the 5’ end of each primer. The dsRNA was synthesized using T7 RiboMAX Express RNAi (Promega, Madison, WI, USA) according to the manufacturer’s instructions, and 23 nL (10 μg/μL) of synthesized dsRNA was precisely injected into the root of the last pair of legs of the ticks using a microinjector (Drummond Scientific, USA). Interference-treated ticks were left to stand for 12 hours (n = 50) and then fed simultaneously with controls (n = 50) on the same mice infected with *B. microti*. Two engorged nymphs were assigned to each group, with at least five biological replicates included, and the weight differences between the control and experimental groups were then statistically compared. In the absence of significant differences, DNA was extracted from the ticks for *B. microti* detection. Given that interference with ferritin 1 affects tick blood feeding, we performed gene silencing of ferritin1 and GPX4 in adult ticks, followed by injection of 0.2 µL of purified *B. microti* suspension into their midguts three days after silencing. Samples were collected 3 days post-injection, with each group containing at least five biological replicates.

### Erastin and ferrostatin-1 treatment

The effects of ferroptosis on *B. microti* infection were investigated using the ferroptosis inducer Erastin (Beyotime # SC0224) and the inhibitor Ferrostatin-1 (Yeasen #54020ES08). Erastin and Ferrostatin-1 were microinjected into engorged nymphs infected with *B. microti* at a volume of 69 nL (Feng et al., 2021). DMSO was used as a control, and samples were collected 24 hours after injection.

### *B. microti* detection

DNA was extracted from the collected samples using a previously reported method that involved first lysing the tissue and then purifying the DNA (Wu et al., 2017). qPCR was subsequently performed against the *B. microti*-specific 18S rRNA gene to detect the presence of pathogens. Briefly, a *B. microti*-positive control was prepared by cloning the 407 bp PCR amplicon into a pMD18-T plasmid to create a standard curve. The qPCR amplifications were conducted in triplicate using a QuantStudio 5 Real-Time PCR machine (Applied Biosystems, Foster City, CA, USA). The PCR system and conditions were based on the Premix Ex Taq™ (TaKaRa, Japan) instruction manual. The reaction mixture consisted of 0.4 μL of each primer, 0.3 μL of the probe, 10 μL of Premix Ex Taq (Probe qPCR), and 3 μL of DNA sample, with a final volume adjusted to 20 μL using water. Primer and probe concentrations were 10 μM (**Supplementary Table S3**). Thermal cycling conditions included an initial denaturation at 95°C for 30 s followed by 40 cycles of denaturation at 95°C for 5 s and 60°C for 34 s. The sample copy numbers were derived from the standard curve and normalized by sample concentration to analyze inter-sample variability.

### Recombinant protein expression, purification, and antibody production

The open reading frames of *HRF*, *ferritin 1*, and *GPX4* genes were cloned into pET-30a using the specific primers listed in **Supplementary Table S1**, and the recombinant proteins were produced in *Escherichia coli*. According to the manufacturer’s instructions, the purified recombinant proteins were isolated using a BeaverBeads™ His-tag Protein Purification system. Polyclonal antibodies were prepared using subcutaneous injection of recombinant proteins in 6-week-old female mice. A 1:1 combination of 100 µg recombinant protein and complete Fuchs adjuvant was used (Sigma, St. Louis, MO, USA) for the initial immunization, and booster immunizations were performed using a 1:1 mixture of 50 μg of protein and incomplete Fuchs adjuvant (Hu et al., 2021). Injections were given every 2 weeks, and serum was collected 10 days after the last immunization and stored at −20°C.

### Western blot

Protein samples were collected from *B. microti*-infected nymphs (engorgement and 2 days post-engorgement, n = 5 each), as well as from uninfected engorged nymphs. Samples were extracted in 500 μL lysis buffer with a protease inhibitor and processed using a tissue grinder. To evaluate the expression of ferritin 1 and GPX4, the ticks were treated with Erastin, Ferrostatin-1, and DMSO. RNAi was performed targeting the HRF gene in nymphs before feeding, with luciferase RNAi as the control, and samples were collected from engorged nymphs for analysis. Proteins were extracted, quantified using the Bradford Assay (Beyotime), run on 12% SDS-PAGE, and transferred to PVDF membranes. The membranes were incubated with primary antibodies (1:200) overnight at 4°C, with alpha-tubulin (1:5,000) as a reference. The secondary antibody (1:10,000) was incubated for 2 hours at room temperature. Protein signals were detected using chemiluminescent reagents.

### Transcriptomic analysis

Three biological replicates were prepared from three engorged ticks treated with *HRF* interference and controlled *Luciferase* interference before blood feeding. RNA was extracted and sent to Majorbio (Shanghai, China) for sequencing and analysis. Eukaryotic mRNA sequencing libraries were constructed, and the raw data were quality-controlled and filtered using fastp (https://github.com/OpenGene/fastp). Trinity software was used for assembly. The assembly involved Inchworm (contig construction), Chrysalis (contig clustering), and Butterfly (generating full-length transcripts). The assembly was optimized with TransRate, and redundant sequences were removed using CD-HIT. Assembly integrity was assessed with BUSCO for quality assurance. Gene and transcript expression levels were quantified using RSEM, differential expression analysis was performed using DESeq2, and genes were considered significantly different using FDR < 0.05 and |log2FC| ≥ 1 criteria. GO functional enrichment and KEGG pathway analyses were conducted using Goatools and Python SciPy software, respectively.

### Detection of divalent iron

The midgut of engorged nymphs was dissected under a stereo microscope, and blood components were removed. Samples were added with Iron Assay buffer and rapidly homogenized with a homogenizer (#MAK025, Solarbio), and after a reaction of 30 min at 25°C, an iron probe was added to continue the reaction at 25°C for 60 min. The absorbance was measured at 593 nm.

### ROS quantification

ROS levels were determined using a 2’,7’-Dichlorodihydrofluorescein diacetate (DCFH-DA) probe according to the instructions provided by Solarbio (#D6470). The midguts of ticks infected with *B. microti* were washed with PBS and incubated with 200 μL of DCFH-DA (5 mM) at 37°C for 60 minutes in the dark. After incubation, the midguts were washed with PBS, placed on slides coated with an anti-fluorescent bursting agent (Sigma-Aldrich, USA), and covered with coverslips. Fluorescence microscopy imaging was performed at an excitation wavelength of 504 nm and an emission wavelength of 529 nm.

### Transmission electron microscopy

Dissected midgut samples were washed three times with PBS into a 1.5 mL centrifuge tube and then fixed with 2.5% glutaraldehyde overnight. The samples were rinsed with 0.1 M PBS (pH 7.4) three times for 15 min each and then fixed with 1% osmium acid (0.1 M PB, pH 7.4) at room temperature for 2 h, avoiding the light. The samples were then sequentially dehydrated with 30% to 100% alcohol for 20 min each, embedded with 100% acetone, and incubated overnight at 37°C. The samples were polymerized on an embedding plate at 60°C for 48 h and then stored as blocks. The embedded samples were sliced with an ultrathin microtome to a 60–80 nm thickness on 150 mesh copper grids, then stained with 2% uranyl acetate for 8 min, washed with 70% alcohol three times, and then rinsed with ultrapure water. The samples were stained with 2.6% lead citrate for 8 min, avoiding CO_2_, washed with ultrapure water, and slightly dried with filter paper. The grids were dried at room temperature for overnight. Images were observed and collected using a HITACHI transmission electron microscope.

### Statistical analysis

Quantitative results were expressed as mean ± standard deviation. The differences between group means were analyzed using a two-tailed Mann-Whitney U test, Student’s t-test, or one-way analysis of variance. GraphPad Prism 6 software (GraphPad Software Inc., San Diego, CA, USA) was used for analyzing the results; p < 0.05 was considered significant.

## Results

### Presence of *B. microti* in *Haemaphysalis longicornis*

**Figure 1:**
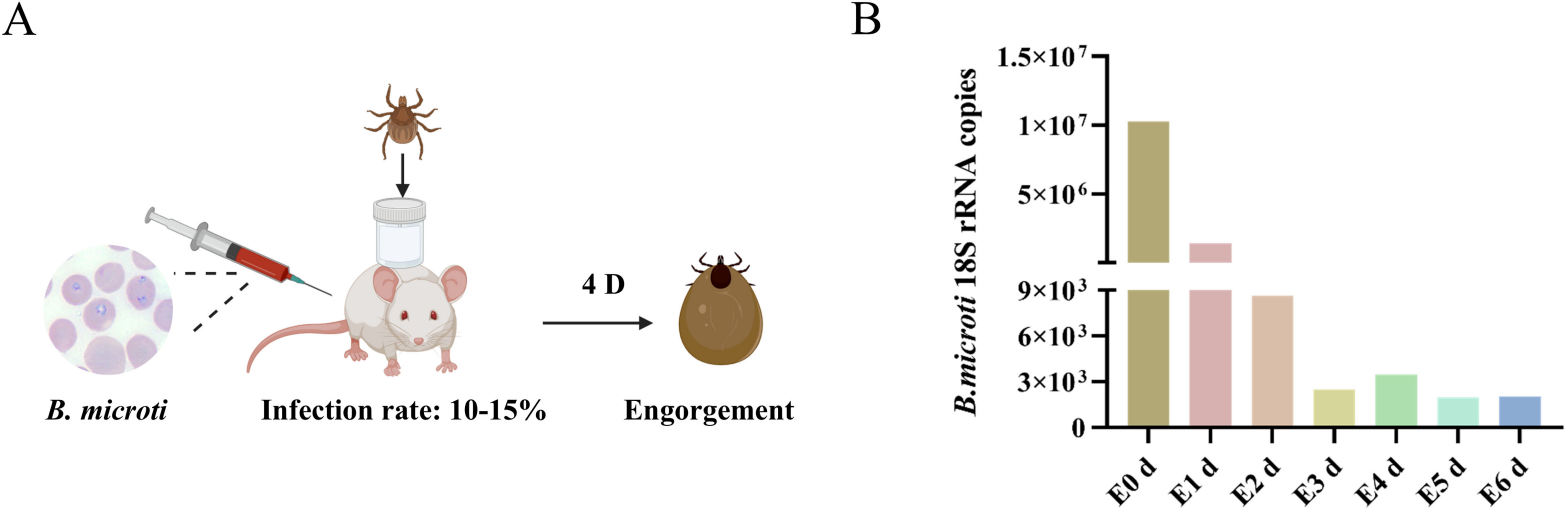
*B*. *microti* presence in the engorged nymph. **(A)** Workflow diagram of *B*. *microti*–mouse–tick infection model. **(B)** *B*. *microti* presence in the engorged nymph.

*B. microti* parasitizes erythrocytes, enters the tick midgut via the bloodstream, develops into male and female gametes, and forms a syncytium that crosses the midgut epithelium into the hemolymph, then invades the salivary glands, and develops into ascospores. When the tick feeds on the next host, these ascospores are released into the host’s bloodstream, completing the life cycle (Jalovecka et al., 2018). We developed a model of *B. microti*-mouse-tick infection based on the life history of *Babesia*. Following the manipulations outlined in **Figure 1A**, we collected tick samples and analyzed *B. microti* present from post-engorgement nymphs using absolute quantitative polymerase chain reaction (qPCR). Three replicates per group were collected, and the experiment was triplicated. Our findings revealed that the abundance of *B. microti* in the tick body peaked after full engorgement, declined during 1–3 d post-engorgement, experienced a slight rebound at 4 d, and then decreased again on days at 5–6 d (**Figure 1B**). These changes may be associated with alterations in the tick’s internal environment following engorgement, the parasite’s metabolic activity, or the tick’s immune response (Cupp, 1991).

### *B. microti* triggers tick ferroptosis

**Figure 2:**
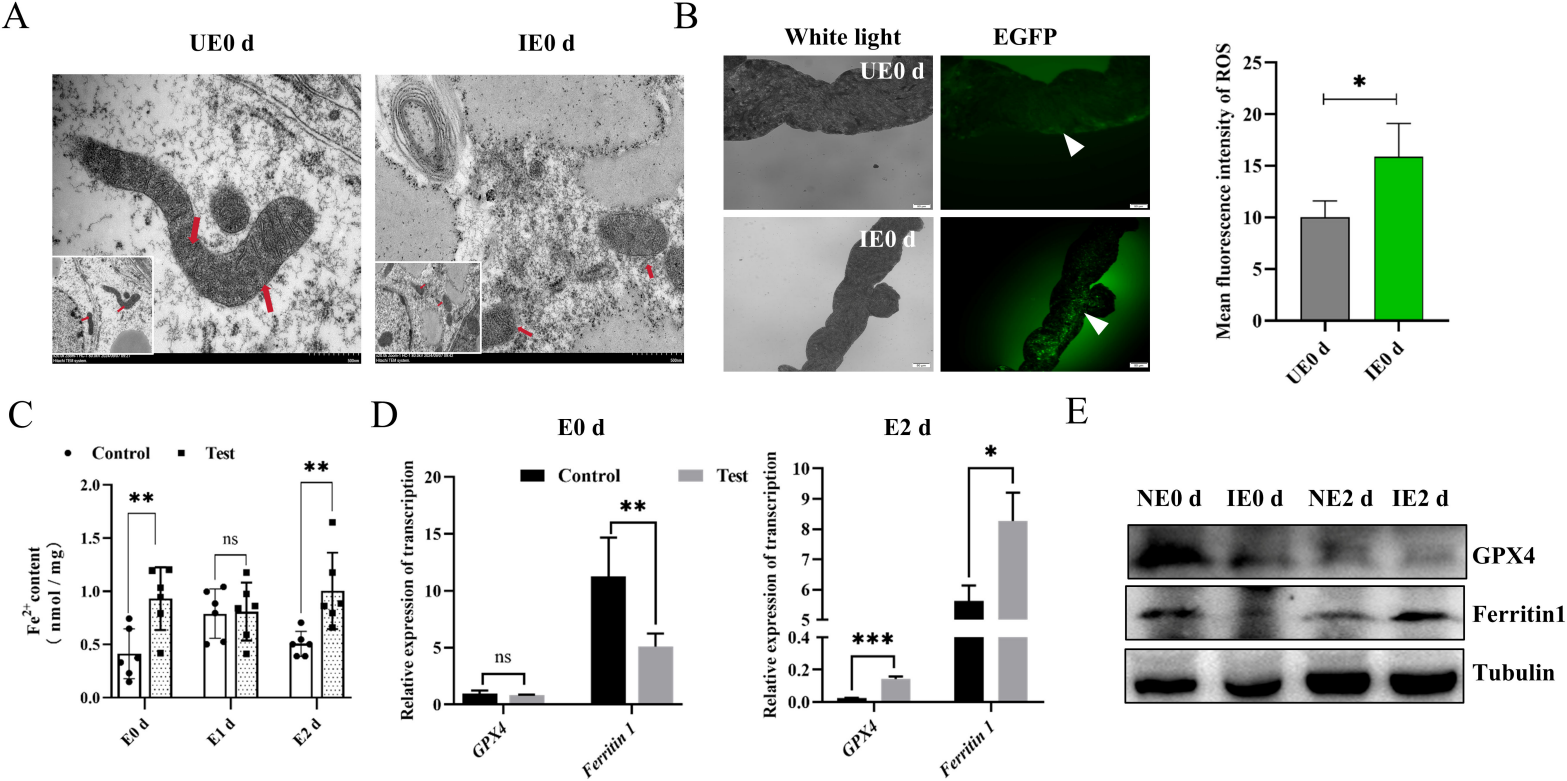
*B*. *microti* promotes tick ferroptosis. **(A)** Transmission electron microscopy detection of the midgut mitochondria of the engorged nymph. Red arrows indicate mitochondria. Scale bar = 500 nm. Magnification, 20,000×. **(B)** Detection of reactive oxygen species in the engorged nymph midgut of ticks (Green). The white arrow points to a green fluorescent light. The quantified fluorescence intensity is presented on the right. Scale bar = 50 μm; Magnification, 20×. **(C)** Detection of Fe^2+^ in the engorged nymph midgut. Quantitative polymerase chain reaction **(D)** and western blot analysis **(E)** of fettirin1 and GPX4 levels in the nymph at 0 d and 2 d post-engorgement. E, engorgement; UE, uninfected *B*. *microti*; IE, infected *B*. *microti*; *P < 0.05, determined using Student’s *t*-test. ns, P > 0.05; *P < 0.05; **P < 0.01; ***P < 0.001; ****P < 0.0001.

Pathogens can promote their reproduction by hijacking the machinery of host cells (Dantas-Torres et al., 2012; Lee et al., 2023). Increased intracellular levels of divalent iron manifest Ferroptosis, increases in ROS, mitochondrial atrophy, reduction of mitochondrial cristae, and rupture of cell membranes (Tang et al., 2021). This process is important in pathogen multiplication, pathogenesis, and immune escape (Gao et al., 2023). Since nymphs have the highest levels of *B. microti* at 0 d post-engorgement, we selected this stage to evaluate the mitochondrial and ROS levels in the tick’s midgut. Transmission electron microscopy (TEM) observations showed that midgut mitochondria in the infected nymphs exhibited reduced volume and the presence of mitochondrial ridges compared with the control group (**Figure 2A**). We found significantly higher levels of ROS in the intestines of engorged nymphs infected with *B. microti* compared with uninfected ticks (**Figure 2B**). Additionally, there were higher levels of divalent iron in the intestines of infected ticks at both engorgement and 2 d post-engorgement stages (**Figure 2C**). Ferritin 1 influences the onset of ferroptosis by regulating intracellular iron ion content, while GPX4 is a key intracellular antioxidant enzyme whose activity and expression level directly determine cellular sensitivity to ferroptosis. We conducted qPCR and western blot analyses for ferritin 1 and GPX4 on *B. microti*-infected nymphs 0 d post-engorgement and 2 d post-engorgement. The results showed that *B. microti* significantly decreased the levels of ferritin 1 and GPX4 at 0 d post-engorgement (**Figure 2E**). However, at 2 days post-engorgement, qPCR analysis revealed that the levels of ferritin 1 and GPX4 were significantly higher in the infected group compared to the uninfected group (**Figure 2D**). These results suggest that *B. microti* may promote tick ferroptosis.

### Ferroptosis promotes tick acquisition of *B. microti*

**Figure 3:**
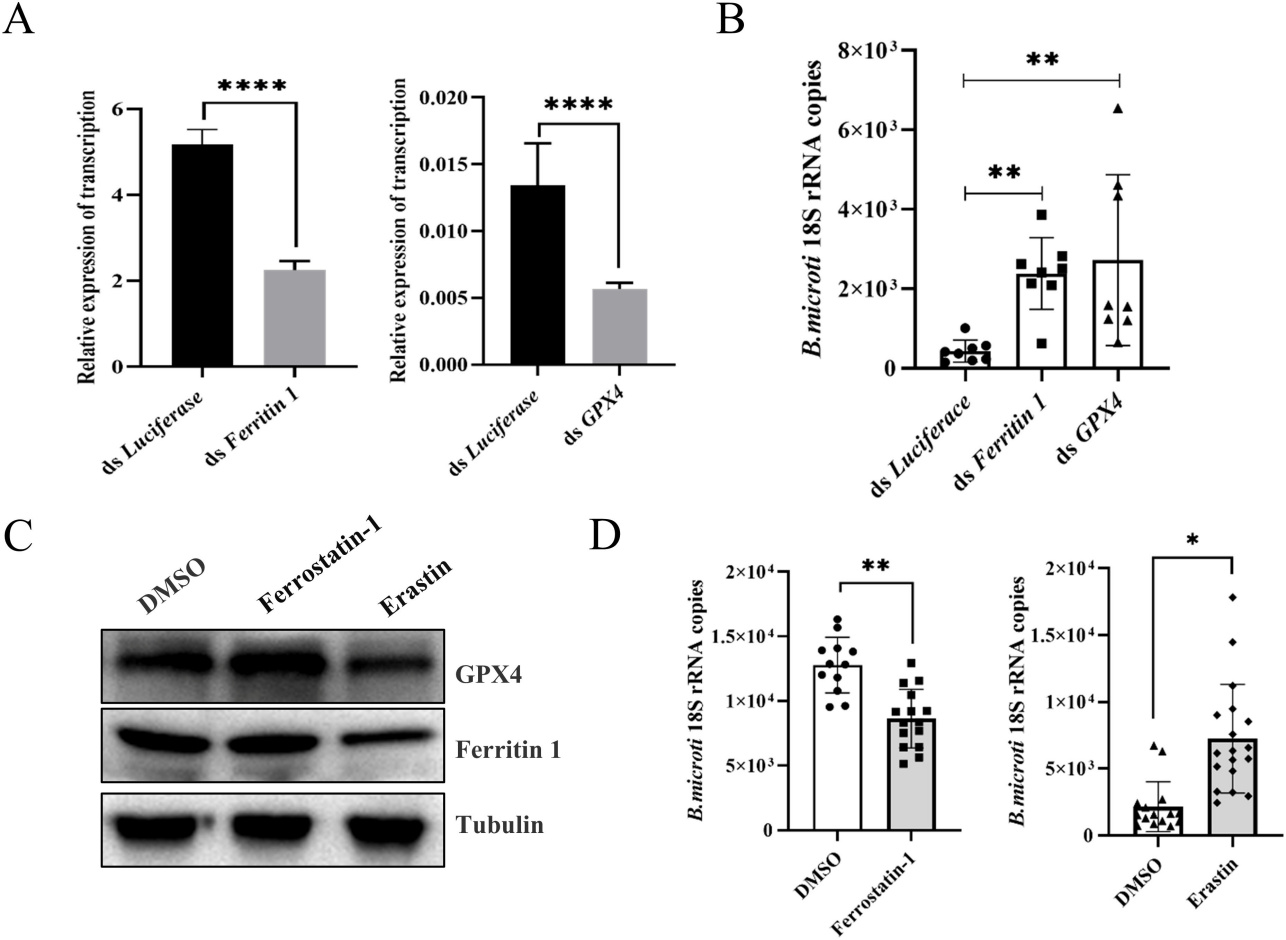
Ferroptosis increased *B*. *microti* load of engorged nymphs. **(A)** Quantitative polymerase chain reaction validation of interference effects. **(B)** Detection of *B*. *microti* load in the adult tick. **(C)** Western blot analysis of engorged nymph ferritin 1 and GPX4 levels 24 h after injection of ferrostatin-1 and erastin. **(D)** A quantitative polymerase chain reaction was used to detect *B*. *microti* load in engorged nymph after the administration of ferrostatin-1 and erastin. *P < 0.05; **P < 0.01; ***P < 0.001; ****P < 0.0001, differential gene expression analysis determined using Student’s *t*-test; *B*. *microti* load analysis determined using two-tailed Mann-Whitney U test.

Erastin promotes ferroptosis by preventing the entry of cystine into the cell, reducing the activity of intracellular GPX4, and increasing the rate of lipid peroxidation (Stockwell and Jiang, 2020). In addition, Erastin can disrupt the mitochondrial permeability transition pores (mPTPs) and accelerate oxidation, thereby accumulating endogenous ROS (Lee et al., 2020). To further investigate the relationship between ferritin 1, GPX4, and *B. microti*, we used RNAi strategy to knock down *ferritin 1* and *GPX4*. Considering that ferritin 1 significantly influences the tick blood-feeding process, we injected purified *B. microti* into the midgut of adult ticks in which *ferritin 1* or *GPX4* had been specifically silenced (**Supplementary Figure S1**) (Pal et al., 2004). The results indicated that the RNAi-treated groups (ds *ferritin 1* and ds *GPX4*) exhibited a significant increase in the presence of *B. microti* (**Figures 3A, B**). Ferrostatin-1 acts as an inhibitor by directly scavenging intracellular lipid peroxides and regulating intracellular iron homeostasis. To clarify the relationship between the occurrence of tick ferroptosis and *B. microti*, we performed *in vivo* experiments using a ferroptosis promoter (Erastin) and an inhibitor (Ferrostatin-1). The expression levels of ferritin 1 and GPX4 were reduced when the ticks were injected with Erastin after engorgement, as verified by Western blot experiments (**Figure 3C**). Ticks injected with Erastin (10 mM) showed an increased *B. microti* load compared to the DMSO-injected group, while those injected with Ferrostatin-1 (40 mM) exhibited a significant reduction (**Figure 3D**).

### RNAi HRF molecules promote tick acquisition of *B. microti*

**Figure 4:**
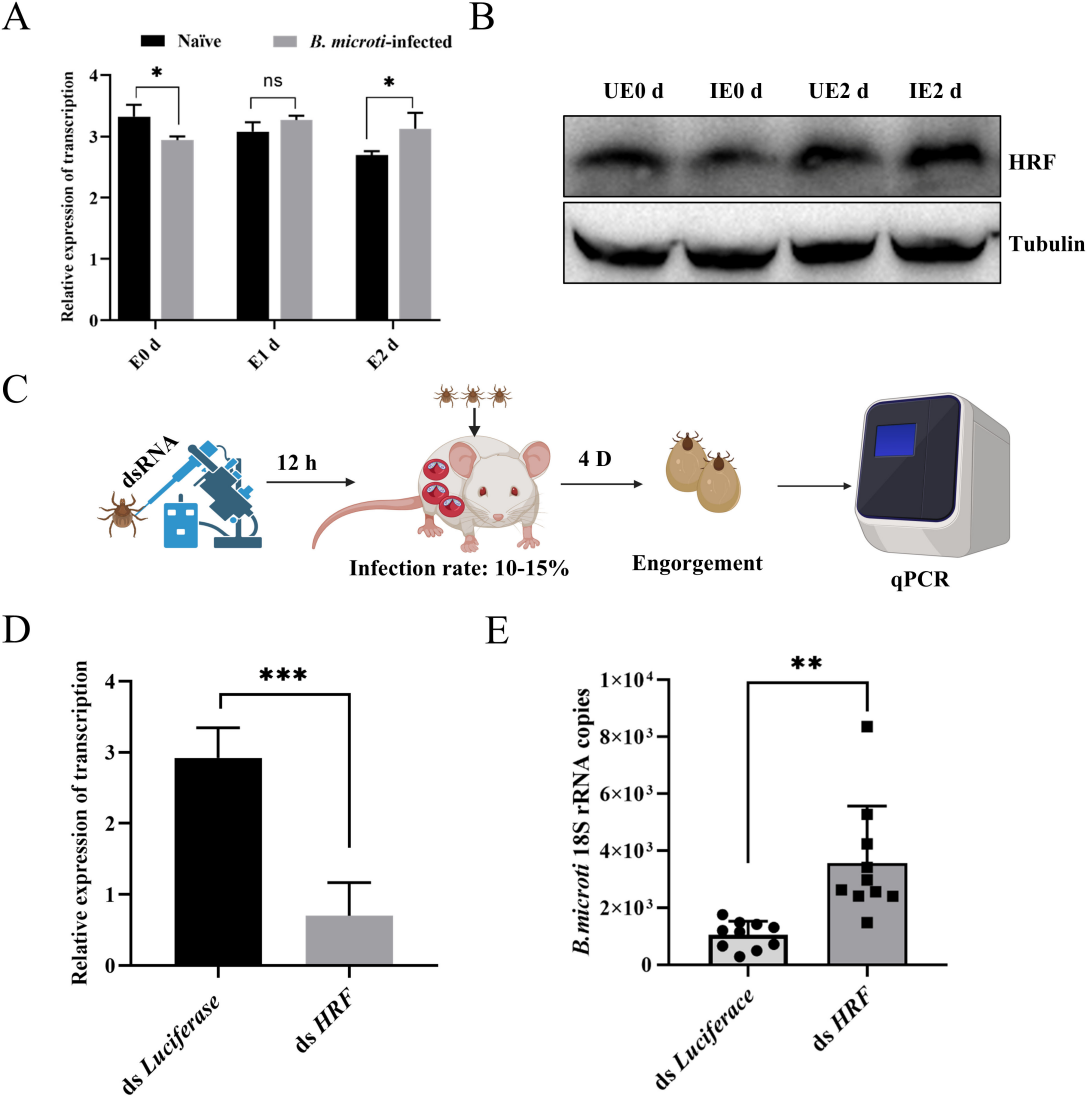
Tick HRF molecules regulated acquisition of *B*. *microti* by nymphs. Quantitative PCR **(A)** and western blot analysis **(B)** of nymph HRF levels from 0 d to 2 d post-engorgement. **(C)** Workflow diagram of interference with nymph and infection with *B*. *microti*. Quantitative polymerase chain reaction validation of interference effects **(D)** and detection of *B. microti* in the engorged nymph **(E)**. *P < 0.05; **P < 0.01; ***P < 0.001; ****P < 0.0001, differential gene expression analysis determined using Student’s *t*-test; *B*. *microti* load analysis determined using two-tailed Mann-Whitney U test.

Tick HRF influences the tick blood-feeding process and pathogen transmission. Previous studies have demonstrated the anti-apoptotic activity of HRF and its function in promoting cell growth and division. Fujise and colleagues have recently proposed that HRF may play a protective role against ROS-induced cell death, where HRF enhances the activities of antioxidant enzymes such as superoxide dismutase (SOD) and glutathione peroxidase (GPx) (Chattopadhyay et al., 2016). Preliminary studies in our laboratory suggest that HRF influences tick ferritin expression, and thus, we hypothesized that *B. microti* affects ferritin expression through HRF. Therefore, we investigated whether HRF affected the tick acquisition of *B. microti*. Engorged and 2 d post-engorgement nymphs were collected from *B. microti*-infected mice and uninfected mice. The infected nymphs showed a decrease in HRF levels at engorgement compared with those fed normal blood, followed by an increase at 2 d post-engorgement, as shown by qPCR analysis (**Figures 4A, B**). Next, we examined the effect of HRF on *B. microti* infection in ticks by using microinjection to interfere with the HRF gene. The ticks were subsequently fed on *B. microti*-infected mice 12 h post-injection. Interference with HRF increased *B. microti* load (**Figures 4C–E**). We microinjected HRF dsRNA into adult ticks, infected them with *B. microti*, and assessed the infection level after 3 d (**Supplementary Figure S2**). *B. microti* load increased in ticks treated with HRF RNAi. These findings suggest that *B. microti* can enhance infection by suppressing tick HRF molecules.

### RNAi Tick HRF molecules promote ferroptosis

**Figure 5:**
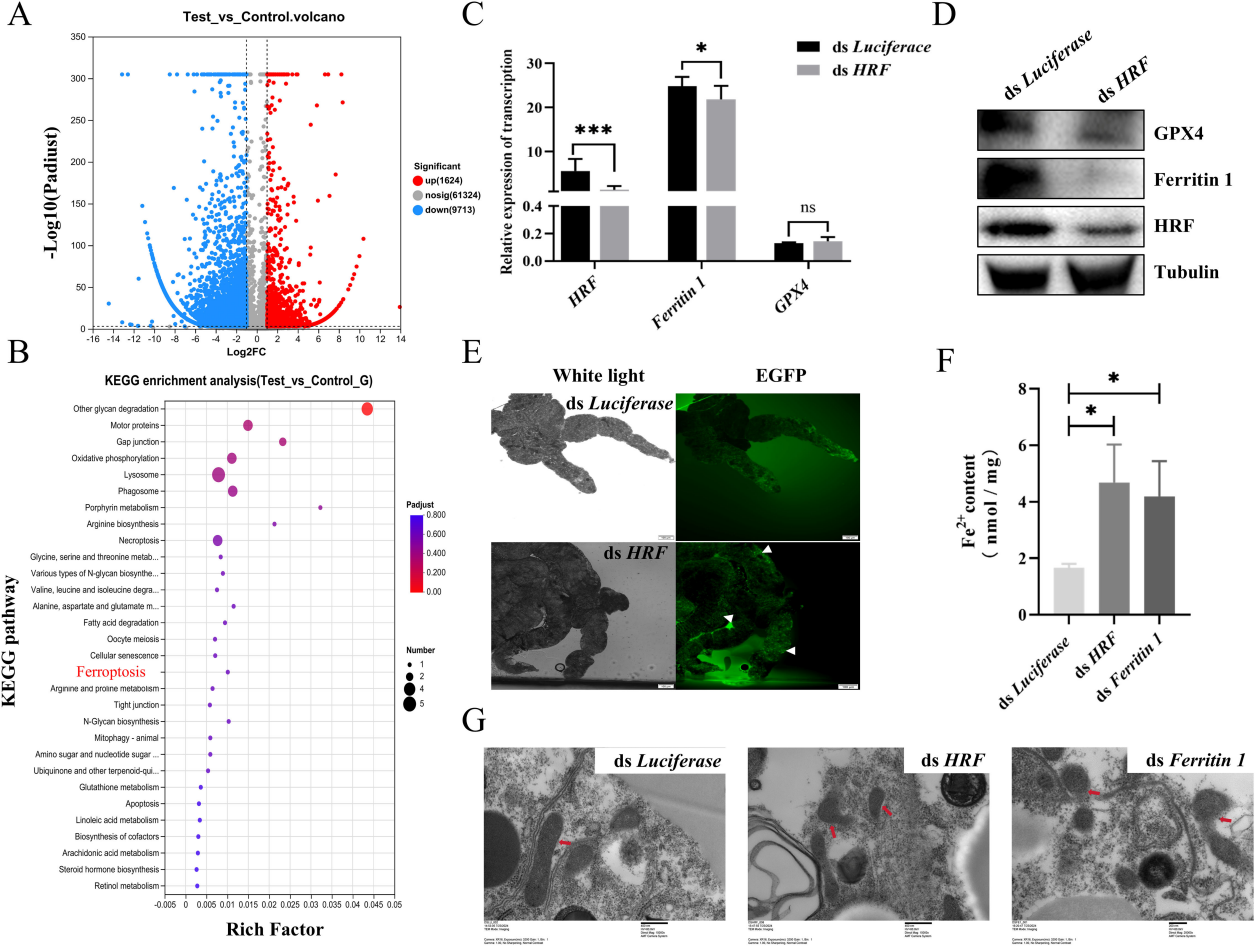
Tick HRF molecules promote ferroptosis. **(A)** Volcano plot showing differentially expressed genes interfering with *HRF* and *Luciferace*. **(B)** Bubble chart displaying 30 pathways related to Kyoto Encyclopedia of Genes and Genomes (KEGG) pathways. Quantitative polymerase chain reaction **(C)** and western blot analysis **(D)** of engorged nymph HRF, Ferritin 1, and GPX4 levels after interfering with *HRF*, determined using Student’s *t*-test. **(E)** Detection of tick midgut ROS after interfering with *HRF*. Scale bar = 100 μm. Magnification, 10×. Detection of tick midgut iron content **(F)** and transmission electron microscopy detection of midgut mitochondrion **(G)** after interfering with *HRF* and *ferritin 1*. *P < 0.05; **P < 0.01; ***P < 0.001; ****P < 0.0001, determined using a one-way analysis of variance. Scale bar = 400 nm. Magnification, 15,000×.

The downregulation of the HRF expression promoted tick acquisition of *B. microti*. Consequently, we further investigated the relationship between HRF and ferroptosis. We performed RNAi targeting HRF in nymphs, then parasitized them to mice infected with *B. microti*, and subsequently performed transcriptome analyses on the engorged ticks. The analysis identified 72,661 mRNAs, of which 11,337 showed differential expression; 1,624 were significantly upregulated, and 9,713 were downregulated (**Figure 5A**, **Supplementary Table S4**). Gene ontology analysis of the interference-treated ticks showed significant enrichment of genes associated with cellular processes, including cell growth, death, transport, and catabolism. Enrichment in cell growth and death was related to ferroptosis (**Figure 5B**). qPCR and Western blots further confirmed that interfering with HRF molecules reduced the ferroptosis-related proteins ferritin 1 and GPX4 (**Figures 5C, D**). Therefore, interfering with HRF downregulates ferritin 1 and GPX4 and promotes ferroptosis.

Next, we explored the relationship between HRF and ferroptosis by examining indicators of ferroptosis associated with interference of HRF in engorged ticks. The degradation or impaired function of Ferritin leads to the release of stored iron into the cell, resulting in elevated intracellular Fe^2+^ concentrations and ROS levels (Mumbauer et al., 2019). ROS activity was higher in the midgut of the RNAi HRF group than in the control group (**Figure 5E**). There were significant increases in the iron content and ROS in the RNAi HRF group (**Figure 5F**). Ferroptosis exhibits distinctive morphological features, with mitochondria appearing shrunken and damaged when viewed under the electron microscope, while the nucleus remains intact. We used TEM to examine mitochondrial morphology after RNAi of tick HRF. In the control group, the structure of the mitochondria remained relatively intact, with no apparent damage, and the mitochondrial ridges were visible. In contrast, most of the mitochondria appeared fragmented, with the inner and outer membranes missing in the experimental group (**Figure 5G**). These results suggest that the downregulation of HRF leads to ferroptosis in ticks.

## Discussion

Ticks can inhibit *Babesia* infection through the action of innate immune-related genes (Antunes et al., 2017; Feng et al., 2024). However, the ticks can still act as disease vectors, suggesting that *Babesia* may promote its infection through other mechanisms that are still poorly understood. This study shows that *B. microti* triggers ferroptosis in ticks by downregulating HRF protein.

HRF has been shown to play an important role in protecting cells from ROS-induced apoptosis by interacting with the antioxidant enzyme peroxidase-1 (PRX1) and masking its phosphorylation site, thereby preventing PRX1 inactivation by the kinase Mst-1 (Chattopadhyay et al., 2016). HRF overexpression enhances peroxidase activity, safeguarding mice against ROS-induced liver injury. The relationship between HRF and oxidative stress in ticks has not been fully characterized. Tick HRF proteins are significantly upregulated after blood feeding and engorgement, playing a role in the synthesis of intracellular Ferritin1 and Ferritin2 (Sun et al., 2024). Ferritin reduces the intracellular concentration of divalent iron, thereby enhancing the antioxidant capacity of the cells, suggesting that HRF may be associated with cellular resistance to oxidative stress. When ticks are infected with *Babesia* during blood feeding, *B. microti* may use tick HRF molecules and their role in combating oxidative stress to facilitate infection. In the present study, we found that *B. microti* could cause dynamic changes in tick HRF molecules upon engorgement, whereas knockdown of the tick HRF gene promoted tick acquisition of *B. microti*. When the tick HRF proteins were knocked down, the intracellular concentration of divalent iron was significantly increased, and this led to an increase in the production of ROS and a decrease in ferritin synthesis. The increase in ROS triggers oxidative stress in cells, and excess Fe^2+^ may promote the Fenton reaction, generating more hydroxyl radicals (-OH) and further exacerbating oxidative damage (Niu et al., 2021). In the RNAi-HRF treatment group, TEM observations of mitochondria in the tick midgut revealed that the inner and outer mitochondrial membranes had either disappeared or decreased in size, whereas the nuclei remained unaffected. This indicates that disrupting the antioxidant function of HRF may facilitate the process of ferroptosis.

Ferritin 1 protects cells primarily by storing iron and reducing oxidative damage from free iron. For example, viruses can reduce intracellular ROS levels by promoting the synthesis of ferritin, a mechanism that can reduce oxidative stress-induced cellular damage, thereby providing a favorable environment for viral replication (Hou et al., 2016; Zhou et al., 2020; Gryzik et al., 2021). In mosquitoes, ferritin knockdown increased ROS levels and inhibited dengue virus infection (Zhu et al., 2019). In our study, we found that *B. microti* increased ROS production by downregulating ferritin levels to facilitate its infection of ticks. Although this finding contrasts with the results of the aforementioned studies, both suggest that pathogens may influence infection by modulating ferritin levels and regulating intracellular ROS production. We hypothesize that the reduced ferritin synthesis may supplement *B. microti* with iron, thereby favoring the growth of the parasite, since iron is an essential nutrient. In addition, *B. microti* affects cells by decreasing ferritin synthesis and increasing ROS levels, thus promoting infection. *B. microti* also downregulates GPX4, a protein that prevents oxidative damage and ferroptosis by reducing the accumulation of lipid peroxides, while *B. microti* increases divalent iron in ticks. TEM assays showed that the mitochondria of the tick midgut displayed diminished ridge structures, and the number of mitochondria was reduced, suggesting that *B. microti* may induce mitochondrial damage in ticks.

In general, viruses or bacteria tend to use ferroptosis cells as sites for replication and release, a process that both exacerbates host cell damage and contributes to the spread of pathogens (Kung et al., 2022; Zhao et al., 2024). For example, Newcastle disease increases iron levels to induce ferroptosis and promote viral replication (Meng et al., 2012). Porcine Reproductive and Respiratory Syndrome Virus can also cause ferroptosis-promoting infections (Mao et al., 2024). Cheng and colleagues found that the H1N1 swine influenza virus induced ferroptosis in an A549 cell line, which enhanced viral replication (Cheng et al., 2022). In this study, Erastin and Ferrostatin-1 were used to promote and inhibit ferroptosis in *B. microti*-infected ticks, and ferroptosis promoted *B. microti* infection. This result was consistent with the knockdown of tick HRF molecules.

Ticks, as disease vectors, have evolved specific mechanisms to defend themselves against potential harm from pathogens. Notably, pathogens frequently exploit ferroptosis as a strategic mechanism to enhance their survival and propagation within host organisms. Infection with *B. microti* has been demonstrated to induce ferroptosis in ticks. However, according to the present study, ferroptosis occurred on the day the ticks were engorged but was significantly suppressed at 2 days post-engorgement. This finding suggests that *Babesia* manipulates tick ferroptosis via HRF and that ticks can counteract ferroptosis through other intrinsic mechanisms to maintain their survival. Ticks acquire *Babesia* by blood feeding, but the number of *Babesia* decreases after tick engorgement and only very few can break through the midgut barrier (Yano et al., 2005). This infection process is likely associated with cell death. *B. microti* can inhibit cell apoptosis at times and activate porin expression for invasion at other times (Zheng et al., 2020). Transcriptomic analysis of *B. microti*-infected ticks identified key biological pathways associated with DNA replication, lipid and small molecule metabolism, and apoptosis-related processes (Feng et al., 2024). In this study, we found that *B. microti* induces tick ferroptosis via HRF proteins. Coupled with findings from a previous study by Nana Wei, which demonstrated that Babesia can disrupt the tick peritrophic matrix (PM) (Wei et al., 2021), we hypothesized that *Babesia* may use cell death to disrupt the tick PM to achieve invasion and evade host immunity. Additionally, the activation of the tick-Toll pathway has been proposed as a mechanism to control infection of ticks by *B. microti*, highlighting the intricate host-pathogen interactions involved in tick-borne diseases (Jalovecka et al., 2024). Nonetheless, there is still a need for an in-depth investigation of how *B. microti* interacts with HRF and how ticks specifically inhibit the mechanistic pathways leading to ferroptosis.

In conclusion, our study demonstrates that *B. microti* infection triggers ferroptosis in ticks, a process characterized by the deregulation of key ferroptosis-related proteins, such as ferritin 1 and GPX4, elevated ROS levels, and impaired antioxidant defense mechanisms. This ferroptosis response facilitates tick acquisition of *B. microti*, highlighting the critical role of ferroptosis in promoting parasite infection. Furthermore, RNAi targeting tick HRF molecules enhances tick acquisition of *B. microti* but induces ferroptosis, underscoring the pivotal function of HRF in regulating this cell death pathway. Collectively, these findings reveal a novel molecular mechanism by which *B. microti* exploits tick ferroptosis to establish infection, providing new insights into the interactions between ticks and *Babesia* and identifying potential targets for controlling tick-borne diseases.

## Data Availability

The datasets presented in this study can be found in online repositories. The names of the repository/repositories and accession number(s) can be found in the article/**Supplementary Material**.
