# Supplementary material for: Tick HRF-dependent ferroptosis pathway to promote tick acquisition of Babesia microti
Source: Front Cell Infect Microbiol. 2025 Mar 12;15:1560152. doi: 10.3389/fcimb.2025.1560152 (PMC11936993; doi:10.3389/fcimb.2025.1560152)
Supplement: Supplementary file 1 [file Table1.docx]

Supplement

Table 1 The primers of PCR

| Gene name | Primer sequence (5’-3’) | Amplicon size (bp) |
| --- | --- | --- |
| HLFerritin1-qF: | CGCATCAACAAGCAGATCAAC | 82 |
| HLFerritin1-qR: | CGTCGTCACGGTCAAAGTAATA |  |
| HLFerritin1-F | CTCCGTCGACAAGCTTGC  ATGAAGTACCAGAACATGCGC | 324 |
| HLFerritin1-R | TGGTGGTGGTGCTCGAGT  TCAGTCGTCTCCTCCG |  |
| GPX4-qF: | GCACGGAGGCAGACATAAA | 125 |
| GPX4-qR: | CGACTGCTTGAGCTTGAGATAC |  |
| GPX4-F: | CTCCGTCGACAAGCTTGC  AGCCACCGTACTTGTTCTG | 576 |
| GPX4-R: | TGGTGGTGGTGCTCGAGT  TCAAAGACGCGAAGCACCAG |  |
| TRF-Q-qF: | CAGCGAGGATTTCACCTTCA | 130 |
| TRF-Q-qR: | GCTACCCAAATAGTCCGCATAG |  |
| TRF-Q-F: | CTCCGTCGACAAGCTTGC  ATGTTGCTCTTCAAGGACAA | 609 |
| TRF-Q-R: | TGGTGGTGGTGCTCGAGT  TTAAAAAAAAATGTGGCACC |  |
| ELF1A-qF： | CGTCTACAAGATTGGTGGCATT | 106 |
| ELF1A-qR： | CTCAGTGGTCAGGTTGGCAG |  |

Note: The horizontal line below the primer represents the homology arm sequence of the PET-30a plasmid.

Table 2 The primers of dsRNA

| Gene name | Primer sequence (5’-3’) | Amplicon size (bp) |
| --- | --- | --- |
| HIHRF dsRNA-S1: | GGATCCTAATACGACTCACTATAGG  AGAGCGGCCTGGACCTGGTG | 402 |
| HIHRF dsRNA-A1: | AGGACATGACACTGGGCC |  |
| HIHRF dsRNA-S2: | AGAGCGGCCTGGACCTGGTG |  |
| HIHRF dsRNA-A2: | GGATCCTAATACGACTCACTATAGG  AGAGCGGCCTGGACCTGGTG |  |
| HIGPX4 dsRNA-S1: | GGATCCTAATACGACTCACTATAGG  ATGCCTGTTCCATCTACG | 424 |
| HIGPX4 dsRNA-A1: | ACGGGTTGTCCATTCCT |  |
| HIGPX4 dsRNA-S2: | ATGCCTGTTCCATCTACG |  |
| HIGPX4 dsRNA-A2: | GGATCCTAATACGACTCACTATAGG  ACGGGTTGTCCATTCCT |  |
| HIFerritin1 dsRNA-S1: | GGATCCTAATACGACTCACTATAGG CCGCATCAACAAGCAGA | 347 |
| HIFerritin1 dsRNA-A1: | CCAGGAAGTCGCACAGC |  |
| HIFerritin1 dsRNA-S2: | CCGCATCAACAAGCAGA |  |
| HIFerritin1 dsRNA-A2: | GGATCCTAATACGACTCACTATAGG CCAGGAAGTCGCACAGC |  |
| Luciferase dsRNA-S1: | GGATCCTAATACGACTCACTATAGG  GCTTCCATCTTCCAGGGATAC | 294 |
| Luciferase dsRNA-A1： | CGTCCACAAACACAACTCCTCC |  |
| Luciferase dsRNA-S2： | GCTTCCATCTTCCAGGGATACG |  |
| Luciferase dsRNA-A2： | GGATCCTAATACGACTCACTATAGG  CGTCCACAAACACAACTCCTC |  |

Note: The horizontal line below the primer represents the joined T7 promoter sequence.

Table 3 The primers of detection of *B. microti*

| Gene name | Primer sequence (5’-3’) | Amplicon size (bp) |
| --- | --- | --- |
| *B.* *microti*-1-F: | AATTACCCAATCCTGACACAGG | 485 |
| *B. microti*-1-R: | TTTCGCAGTAGTTCGTCTTTAACA |  |
| *B. microti*-2-F: | GACACAGGGAGGTAGTGACAAGA | 407 |
| *B. microti*-2-R: | CCCAACTGCTCCTATTAACCATTAC |  |
| *B.microti* -qF: | AACAGGCATTCGCCTTGAAT | 104 |
| *B.microti* -qR: | CCAACTGCTCCTATTAACCATTACTCT |  |
| Probe | FAM-CTACAGCATGGAATAATGA-MGB |  |
