# Supplementary material for: Tick HRF-dependent ferroptosis pathway to promote tick acquisition of Babesia microti
Source: Front Cell Infect Microbiol. 2025 Mar 12;15:1560152. doi: 10.3389/fcimb.2025.1560152 (PMC11936993; doi:10.3389/fcimb.2025.1560152)
Supplement: Supplementary file 2 [file Table2.docx]

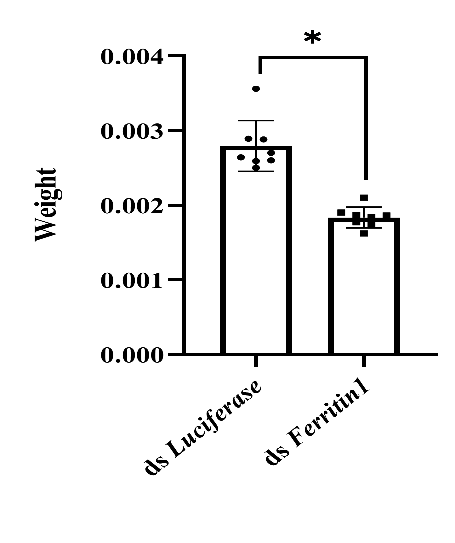


B

A


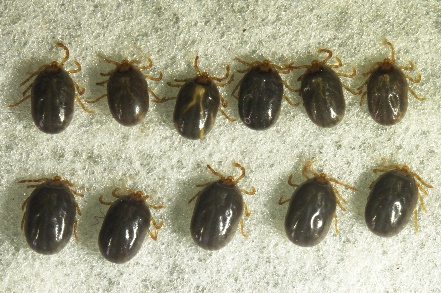


ds *ferritin1*

ds *Luciferase*

Fig. 1. Interference with the effect of *ferritin1* on blood-feeding by the nymph. A Comparison of the size of engorged ticks with controls after interfering with *ferritin 1*. B Statistical plot analysis of interfering *ferritin 1* compared with control weight.


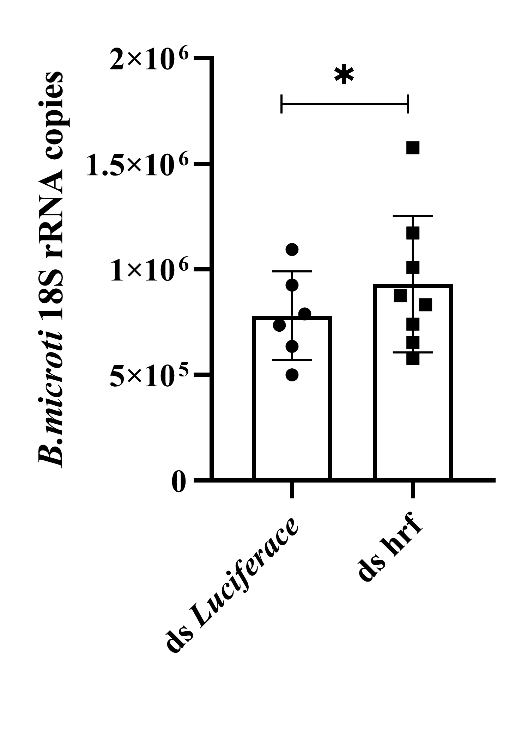


Fig. 2. Analysis of differences in *B. microti* infection in adult tick after interfering with HRF via injection infection.
